# Detection Threshold Estimates for InSAR Time Series: A Simulation of Tropospheric Delay Approach

**DOI:** 10.3390/s21041124

**Published:** 2021-02-05

**Authors:** Emre Havazli, Shimon Wdowinski

**Affiliations:** 1 Rosenstiel School of Marine and Atmospheric Sciences, University of Miami, Miami, FL 33149, USA; 2 Department of Earth and Environment, Institute of Environment, Florida International University, Miami, FL 33199, USA

**Keywords:** InSAR, SBAS, tropospheric delay, data simulation, Socorro Magma Body

## Abstract

We present a method for estimating the detection threshold of InSAR time-series products that relies on simulations of both vertical stratification and turbulence mixing components of tropospheric delay. Our simulations take into account case-specific parameters, such as topography and wet delay. We generate the time series of simulated data with given intervals (e.g., 12 and 35 days) for temporal coverages varying between 3 and 10 years. Each simulated acquisition presents the apparent noise due to tropospheric delay, which is constrained by case-specific parameters. As the calculation parameters are randomized, we carry out a large number of simulations and analyze the results statistically and we see that, as temporal coverage increases, the amount of propagated error decreases, presenting an inverse correlation. We validate our method by comparing our results with ERS and Envisat results over Socorro Magma Body, New Mexico. Our case study results indicate that Sentinel-1 can achieve ≈1 mm/yr detection level with regularly sampled data sets that have temporal coverage longer than 5 years.

## 1. Introduction

Interferometric Synthetic Aperture Radar (InSAR) time-series techniques are important tools for studying tectonic and non-tectonic ground deformation over large areas with millimeter-per-year-level accuracy [1,2,3]. However, InSAR accuracy is affected by various error sources including orbital errors [1], topographic residuals [4,5], phase unwrapping errors [6,7,8], phase decorrelation [9], and tropospheric phase delay (e.g., [10]). An improvement in processing methods and increasing acquisition frequency has reduced the effects of most error sources. Nevertheless, tropospheric delay remains a significant error source in InSAR time series, which propagates through the time-series inversion into analysis products, e.g., velocity field [3,11,12,13,14].

Tropospheric phase delay has been investigated by several authors employing a variety of methods. Several studies have divided tropospheric error into systematic and stochastic components and studied them in space [3,15] and time [10,16,17]. Early methods were based on spatiotemporal analysis of InSAR data alone [18,19,20,21]. More advanced methods used additional data sources for estimating the tropospheric phase delay and removed it from InSAR observations. These additional data sources vary from GPS-derived phase delays [22,23] to satellite-observed phase delays, such as medium resolution imaging spectrometer (MERIS) and moderate resolution imaging spectroradiometer (MODIS) [24,25,26,27,28], and numerical weather models, such as ERA5 of the European Centre for Medium-Range Weather Forecasts (ECMWF) [26,29,30]. All these methods aimed to estimate error due to tropospheric phase delay in InSAR products. The methods above focused on the removal or correction of tropospheric errors in InSAR data, but interferograms did not quantify the total effect of tropospheric delay on the InSAR time series. It is important to have a method that quantifies the signal threshold below which signals due to tropospheric can be distinguished from real deformation signals.

In this study, we investigate and quantify the effect of tropospheric phase delay using a simulation approach based on separation of the tropospheric phase delay into its vertical stratification and turbulence mixing components. We modeled vertical stratification by using a digital elevation model (DEM) and standard deviation of wet delay calculated from precipitable water vapor (PWV) data collected by the MODIS instrument on the Terra and Aqua satellites [31,32]. Turbulence mixing was modeled using a spectral analysis approach following the formulations described in [3]. We carried out our simulations in three stages: first, we simulated vertical stratification; second, we simulated turbulence mixing; and finally, we simulated the combination of both components. Our simulations relied on the assumed range of parameters in each case study and did not include any deformation signal. The exclusion of a deformation signal brought our expected velocity value to 0, which ensured that the obtained velocities were residuals due to tropospheric delay.

Our method can be applied to different regions with different climatic and topographic characteristics. The results presented in this study are specific to the area around Socorro Magma Body. The topography of the study area can be defined as mostly flat with localized prominent changes, while the climate conditions are defined as a mix of arid and semiarid [33]. Application of our method in different topographic conditions such as rapid altitude changes in short distances will result in a higher impact from vertical stratification. A study area with different climate conditions such as tropical and subtropical climate conditions will produce results with a higher impact from turbulence mixing. Our method provides a way to analyze the impact of tropospheric delay in InSAR time series by using a simulation of both components individually while taking different climate and topography conditions into account and incorporating MODIS data for realistic solutions.

## 2. Tropospheric Phase Delay

Variations in tropospheric delay have been recognized as a major source of error in InSAR studies since the early days of the technique [11,12,13]. A methodology to measure tropospheric delay with comparable accuracy, spatial and temporal resolution for InSAR, and complete removal of this effect has not yet been possible. Tropospheric phase delay is caused by variations in water vapor, pressure, and temperature contents in the spatial and temporal domains [3,34,35]. These variations have a direct impact on Synthetic Aperture Radar (SAR) acquisitions, where they reveal themselves as systematic and stochastic errors. These systematic and stochastic errors consist of vertical stratification and turbulence mixing, respectively. Vertical stratification is a systematic error that is correlated with topography and is driven by changes in the refractive index of the tropospheric layers [36]. Turbulence mixing is a process driven by water content motion in the lower troposphere and is governed by fluid dynamics laws [3,14,35].

Early methods for the removal of tropospheric errors relied on the use of spatiotemporal filtering [20,21] or averaging out by using a large number of acquisitions [18,19]. Proposed methods for the removal of tropospheric delay by analyzing only InSAR observations have shown that it is not possible to measure refraction from acquisitions. Using additional data from GPS-derived tropospheric delay maps is not applicable everywhere since there may or may not be enough stations to produce such a map or large distances between GPS stations. Nowadays, the most common method is to use a numerical model. However, numerical methods have a lower spatial resolution in both the horizontal and vertical directions and do not represent the state of the troposphere at the time of acquisition. In light of the shortcomings of correction methods, it is important to estimate an expectation value for the amount of tropospheric delay concerning temporal coverage of our data set.

### 2.1. Vertical Stratification

**Figure 1:**
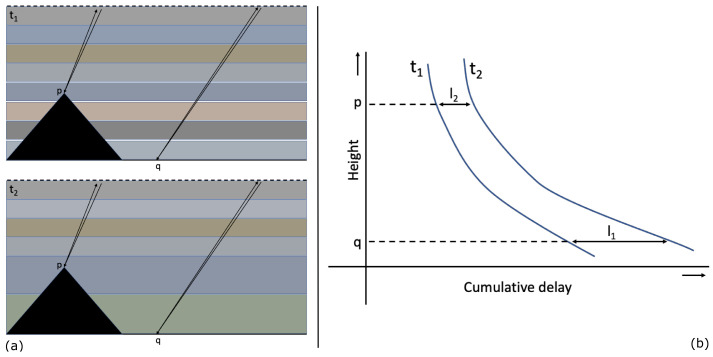
(**a**) Differential tropospheric delay between point *p* at a higher altitude than point *q* with different refractivity profiles at times $t_{1}$ and $t_{2}$, and (**b**) cumulative delay at times $t_{1}$ and $t_{2}$ between points *p* and *q*: the differential delay between between points at different altitudes are determined by the difference between $l_{1}$ and $l_{2}$. Figure adapted from [3].

Tropospheric delay due to vertical stratification is caused by changes in refractivity along the vertical. Regardless of changes in refractivity between two acquisitions, there would be no difference in delay over a flat terrain since image-wide biases do not have an impact on interferograms [3]. Vertical stratification could cause differential delay contributions where the topography of a study area is characterized by significant changes such as hills or mountains. Previous studies showed that the use of elevation-dependent polynomials is an effective tool to determine the effect of vertical stratification [16,37,38]. In the Differential InSAR (DInSAR) method, phase delay due to vertical stratification unfolds as the differential delay between two acquisitions (Figure 1). Therefore, we simulate only the differential delay, which varies in magnitude but correlates with local topography. We assume a linear relationship between topography and vertical stratification, which may not cover all cases but provides a good starting point for our simulations.

### 2.2. Turbulence Mixing

As opposed to vertical stratification, turbulent mixing is not directly correlated with topography and affects both flat terrains and higher altitude terrains. Turbulence mixing is driven by the turbulent flows of water vapor in the lower troposphere and, therefore, can be approximated by complex mathematical methods. Kolmogorov power law, also known as the Kolmogorov length scale, assumes that small-scale turbulent motions are statistically isotropic in three dimensions and puts forward a power spectrum [39]. The structure function of this power spectrum (Equation (1)) explains the motion of turbulent flows in three different length scales.

(1) $$P_{\phi }\left(f\right)=\left\{\begin{array}{cc} P_{I}{\left(f/f_{0}\right)}^{-5/3} & \;\mathrm{for}\;1.5\leq (f_{0}/f)<50\;\mathrm{km},\\ P_{0}{\left(f/f_{0}\right)}^{-8/3} & \;\mathrm{for}\;0.25\leq (f_{0}/f)<1.5\;\mathrm{km},\\ P_{III}{\left(f/f_{0}\right)}^{-2/3} & \;\mathrm{for}\;0.02\leq (f_{0}/f)<0.25\;\mathrm{km}. \end{array}\right.$$

where $P_{\phi }$ is the power spectrum and *f* is the wavenumber in cycles/km. $P_{I}$, $P_{0}$, and $P_{III}$ represent different regimes of the power spectra. Hanssen, 2001, carried out tests with interferograms generated from European Remote Sensing (ERS) acquisitions and showed that the structure function given in Equation (1) can be used to model the effect of turbulence mixing in InSAR studies.

## 3. Data Simulation and Analysis

**Figure 2:**
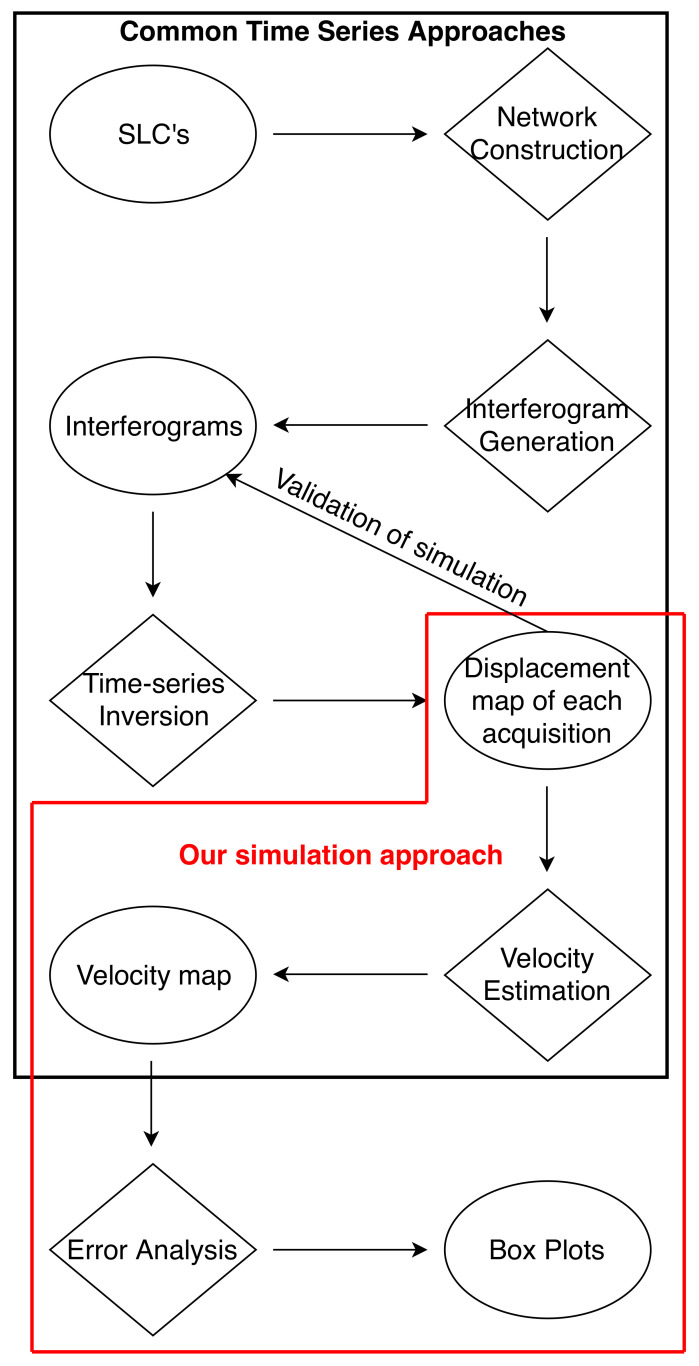
Flow chart showing the standard InSAR time-series process and our simulation method (outlined with red): the diamonds represent processing stages, and the ellipses represent products.

Our data simulation and analysis processes include the following steps: simulation of the time-series epochs, velocity estimation, and error analysis (red frame in Figure 2). The term time-series epoch represents the total displacement map, inverted from corresponding interferograms, for a given acquisition date. The time-series epoch is referenced to a pixel and an acquisition date. For a given time series, each epoch is referenced to the same pixel and acquisition date so that spatial and temporal data unification is achieved. In a regular application of the Small Baseline Subset (SBAS) time series, we expect signals from multiple sources to be present in the time-series epochs (see Appendix A). Since we want to quantify the amount of error caused by tropospheric delay, we assumed no input from other error sources and deformation. It is important to note that the simulation of time-series epochs is preferable over the simulation of interferograms or Single Look Complex (SLC) data. Simulating epochs ensures that we have total control over the signals going into the velocity estimation step and that the obtained results are free from processing errors.

The tropospheric delay varies in both the space and time domains and, therefore, requires a complex approach to capture its total impact. The dynamic nature of the troposphere is commonly separated into two components; the first one is the systematic component, vertical stratification, and the second one is the stochastic component, turbulence mixing. We simulated both components individually to be able to quantify and illustrate their characteristics while isolating them and ensuring that they do not influence each other. We introduced extra multipliers to our simulations to be able to achieve both spatial and temporal variation. The introduced multipliers were randomly selected from a given set, which represented spatial and temporal variation characteristics of tropospheric delay over our study area. Another important aspect impacting a velocity field is the number of epochs that goes into time-series inversion. Since InSAR satellites follow a regular orbit, their revisit time is also regularized, and therefore, we know the maximum number of acquisitions for any given temporal coverage. We simulated epochs for the temporal coverages between 3 years and 10 years, 100 times for each period, to be able to statistically quantify the total errors in final velocity products. We discuss the details of simulation of vertical stratification in Section 3.1.1 and turbulence mixing in Section 3.1.2. We conducted three types of delay simulations. First, we simulated the effect of vertical stratification, then turbulence mixing, and finally the combined effect of both components. Simulating each component individually allowed us to consider the significant characteristic of each component (such as spatial distribution) and to evaluate its effect on the final results quantitatively.

In the velocity estimation step, we used the simulated time-series epochs and carried out a pixel-wise linear regression to calculate the corresponding velocity for each pixel. The details of the velocity estimation step are discussed in Section 3.2. In the final error analysis step, we analyzed the resulting velocity fields by concatenating corresponding temporal coverages together and by calculating the distribution of delay values within the velocity map using histograms. We discuss the details of error analysis in Section 3.3.

### 3.1. Tropospheric Delay Simulations

Tropospheric delay due to vertical stratification is caused by changes in the pressure and temperature gradients of precipitable water. Vertical stratification could cause differential delay contributions, where the topography of a study area is characterized by significant changes. Previous studies showed that the use of elevation-dependent polynomials is an effective tool to determine the effect of vertical stratification [16,37,38]. In the Differential InSAR (DInSAR) method, phase delay due to vertical stratification unfolds as the differential delay between two acquisitions (Equation (A7)). Therefore, we simulated only the differential delay, which varied in magnitude but correlated with local topography. We assumed a linear relationship between topography and vertical stratification that may not cover all cases but provides a good starting point for our simulations. As opposed to vertical stratification, turbulent mixing was not directly correlated with topography. Turbulence mixing was driven by the turbulent flows of water vapor in the lower troposphere. Hanssen (2001) studied the subsets of sample ERS interferograms and showed that the rotational average of their spectrum is in agreement with the Kolmogorov power law at certain wavelengths [3].

Throughout the manuscript, we simulated deviatoric tropospheric delay for each acquisition, which represents the apparent deformation or tropospheric noise in deformation studies. By using deviatoric tropospheric delay rather than deviatoric tropospheric phase delay, the simulations are independent of the sensor wavelength and can be applied to all sensors. If needed, the simulated deviatoric tropospheric delay can be converted to phase delay by dividing its value by the wavelength of the sensor (see Appendix A). We started our tests by simulating delays with the acquisition interval and incidence angle standards of ERS and Envisat satellites. We used a 35-day temporal baseline and projected the simulated scenes onto a 23-degree incidence angle. The ERS and Envisat satellites have been widely used in many scientific studies (e.g., [10,40]) and were selected for comparison with our case study over Socorro Magma Body.

#### 3.1.1. Tropospheric Delay Due to Vertical Stratification

**Figure 3:**
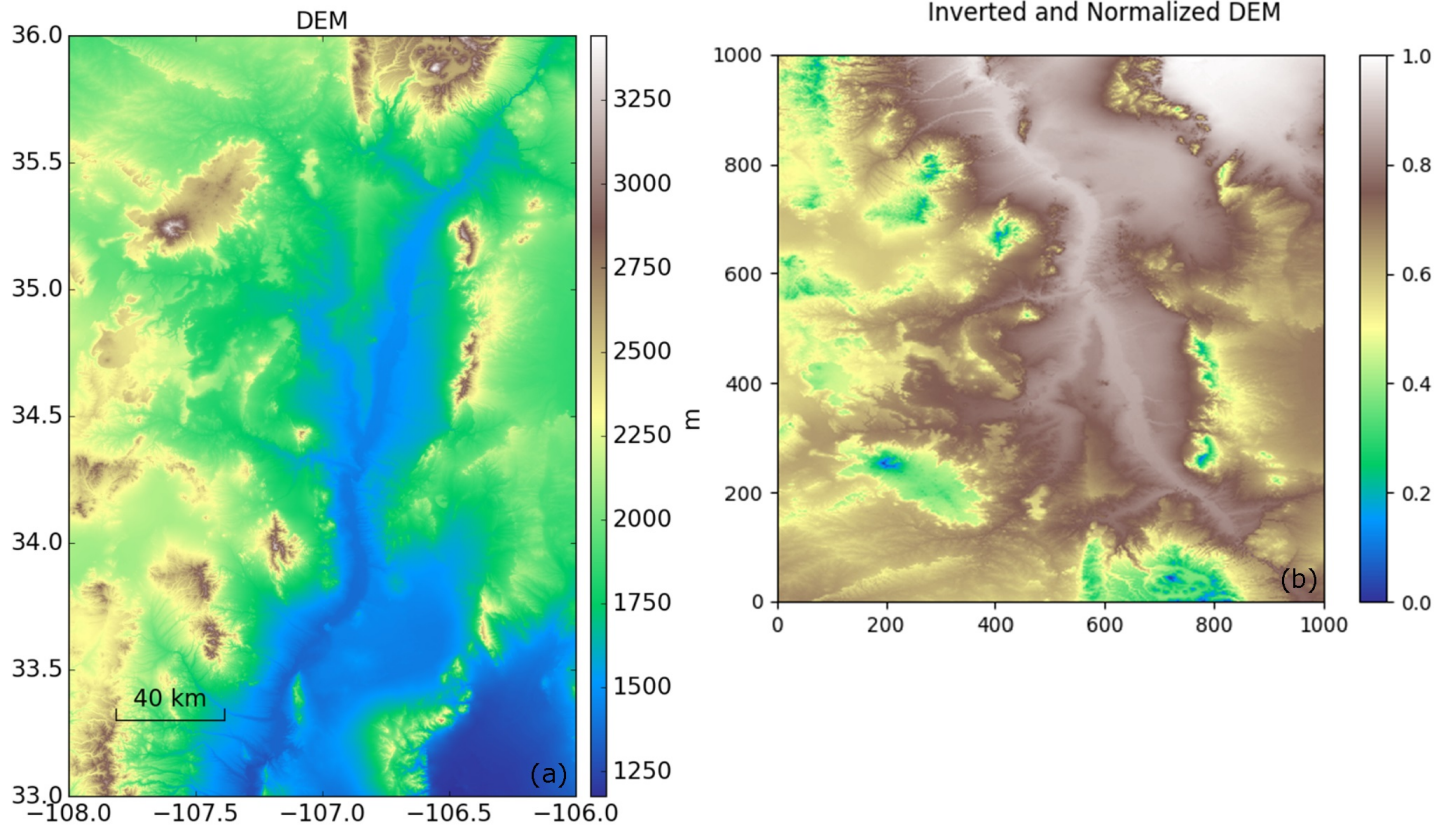
(**a**) The digital elevation model (DEM) of the Socorro Magma Body located in New Mexico, USA, from the Shuttle Radar Topography Mission (SRTM), with 1 arc-second (≈ 30 m) resolution and (**b**) an inverted, normalized, flipped, and resampled DEM used to compute the phase delay due to vertical stratification.

We simulated the contribution of vertical stratification by using topographical information on and around Socorro Magma Body (SMB). We imported the Shuttle Radar Topography Mission (SRTM) digital elevation data with the highest publicly available resolution of 1 arc-second to achieve the spatial correlation of a stratified troposphere (Figure 3a). First, we multiplied DEM heights ($h_{dem}$) with −1 to obtain higher values for lower altitudes and lower values for higher altitudes and normalized them to obtain a unitless surface (*D*):

(2) $$D=\frac{\left(h_{dem}\times -1\right)-{\left(h_{dem}\times -1\right)}_{min}}{{\left(h_{dem}\times -1\right)}_{max}-{\left(h_{dem}\times -1\right)}_{min}}$$

The obtained surface was resampled to form an image with 1000 × 1000 pixels for computational simplicity. We flipped surface $D_{resampled}$ vertically to resemble the descending orbit acquisitions (Figure 3b). Finally, we multiplied surface $D_{resampled}$ with a randomly chosen constant (*m*) from a normally distributed set with 3 cm standard deviation centered at 0 and obtained a single epoch representing vertical stratification delay ($d_{strat}$):

(3) $$d_{strat}=D_{resampled}\times m$$

**Figure 4:**
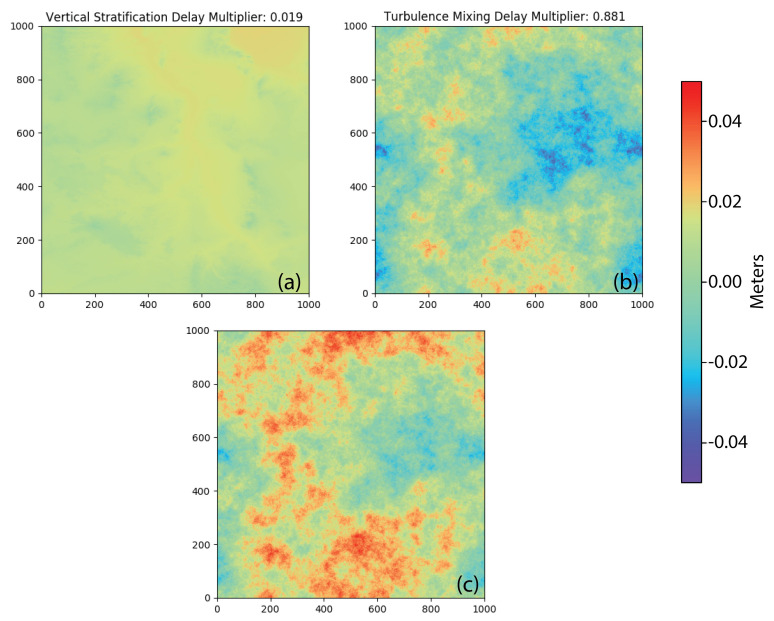
Simulated maps of tropospheric delay: (**a**) vertical stratification delay calculated with random multiplier 0.019, (**b**) turbulence mixing delay calculated with random multiplier 0.881, and (**c**) the resulting delay from the combination of vertical stratification (**a**) and turbulence mixing (**b**) delays.

We selected the standard deviation of our set relying on the absolute standard deviation of slant wet delay calculated from 2939 MODIS PWV observations between 1 January 2002 and 31 December 2012 over our case study area (see Appendix B). This value is specific to our case study area, as described in Section 6. Using the results in other study areas requires a similar analysis of the MODIS PWV data. An example of stratified delay is shown in Figure 4a, which exhibits a small amplitude of deviatoric delay for the value of *m* (0.019). The largest delays are in areas of low elevation, and the smallest delays are in areas of high elevation.

#### 3.1.2. Tropospheric Delay Due to Turbulence Mixing

We adopted the turbulence mixing model defined by Hanssen (2001) (Equation (1)). To translate the dimensionless surface generated by Hanssen’s method to delay in our simulation, we needed to scale it to length and to vary it from one acquisition to another to reflect temporal variability in the magnitude of the delay. Turbulence mixing is random in space by nature, and its effect on InSAR data can vary from a few millimeters to a few centimeters over the same area [3]. We multiplied our simulated surface ($d_{sim}$) with a random parameter (*n*), which varies in the range 0 to 0.1 (Equation (4)), and obtain a simulated epoch representing turbulence mixing delay ($d_{turb}$):

(4) $$d_{turb}=d_{sim}\times n$$

Consequently, the simulated delay varies randomly between 0 and several centimeters, reflecting turbulent delay conditions between different acquisitions. One of the main differences between turbulence mixing and vertical stratification is that turbulence mixing varies in spatial distribution as well as in amplitude whereas vertical stratification varies only in amplitude for each acquisition (Figure 4b).

#### 3.1.3. Tropospheric Delay Due to Combination of Vertical Stratification and Turbulence Mixing

The combined phase delay (Figure 4c) was simulated by adding individually simulated vertical stratification phase delay ($d_{strat}$) (Figure 4a) and turbulence mixing phase delay ($d_{turb}$) (Figure 4b). The resulting image exhibits the delay characteristics of both components. The combined delay image is similar to the turbulence mixing component because of the difference between the vertical stratification (0.019) and turbulence mixing (0.881) multipliers.

### 3.2. Velocity Estimation

The common approach in InSAR studies for obtaining time-series products can be divided into four major steps: network construction, interferogram generation, time-series inversion, and velocity estimation (Figure 2). The data that underpin this approach are in the Single Look Complex (SLC) format, commonly provided by most space agencies. Network construction is based on either single or multiple master approaches. The single master approach produces all interferograms using the same master acquisition. The multiple master approach forms pairs of images by using temporal and perpendicular baseline constraints. Interferogram generation is carried out by cross multiplication of the master image and the complex conjugate of the slave. After the interferograms are calculated, time-series inversion is carried out. The two most common time-series methods are the Persistent Scatterer Interferometry (PSI) [20,41,42] and SBAS techniques [21,43]. PSI adopts a single master approach and relies on the identification of coherent pixels over long time intervals. The SBAS method uses multiple master acquisitions and phase information of unwrapped interferograms to invert for the cumulative Line of Sight (LOS) displacement for each pixel.

In this study, we modified the common SBAS approach, treated our simulated data as time-series epochs, and immediately started from velocity estimation (Figure 2). We skipped the initial three steps of network construction, interferogram generation, and time-series inversion to avoid attendant errors, mainly due to unwrapping. We verified our approach by using the simulated epochs to calculate interferograms and to process them with regular SBAS procedures using the MintPy software package [44]. The validation procedure yields minor differences between our approach and the standard SBAS processing chain, which are attributable to unwrapping errors in the SBAS processing chain. Therefore, our proposed method ensures that our results are not affected by unwrapping errors. We simulated temporal coverages ranging from 3 years to 10 years to observe the relative change in propagation of tropospheric errors for the total observation period.

We did not assume any signal other than the tropospheric delay and, hence, simply treated the simulated data as time-series epochs. We referenced all epochs to the same datum in space and time. The spatial datum was defined and achieved by referencing each epoch to the same pixel. We used the same reference pixel for each epoch and subtracted its value from the rest of the pixels. A temporal datum was achieved by selecting a master date and by subtracting each epoch from the master date. The resulting data set carries the characteristics of a regular time-series stack and can be used for velocity estimation. Velocity estimation was carried out for each pixel by calculating the slope of the best fitting line to time-series epochs.

### 3.3. Error Analysis

**Figure 5:**
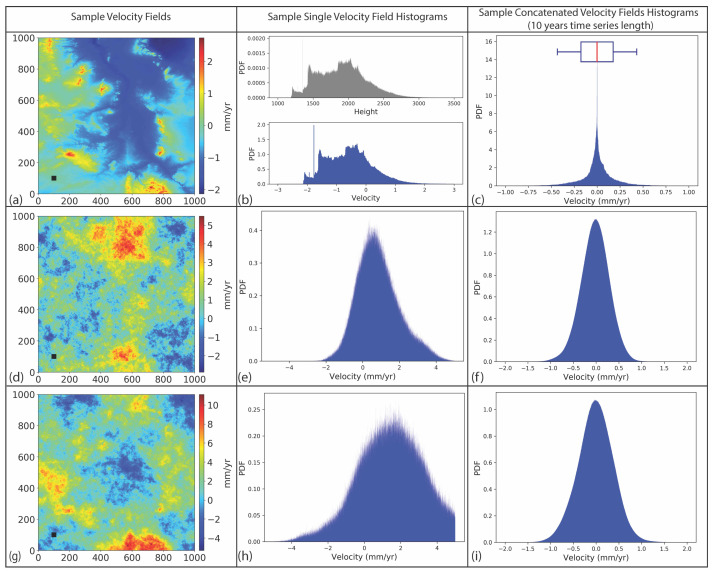
Velocity maps of tropospheric delays due to (**a**) vertical stratification, (**d**) turbulence mixing, and (**g**) their combination tropospheric delays: each velocity map was calculated from 32 simulated scenes with a 35-day acquisition interval reflecting a 3-year-long time series. (**b**) A histogram representing the velocity distribution in (**a**) compared with elevation distribution. (**c**) The velocity distribution obtained by joining 100 tests of random vertical stratification. The box plot represents a 95% confidence interval. (**e**,**h**) Histograms representing the velocity distribution in (**d**,**g**), respectively. (**f**,**i**) Histograms obtained by joining 100 tests of turbulence mixing (**d**) and a combination of vertical stratification and turbulence mixing (**g**).

We did not introduce any deformation signal, and consequently, the calculated velocity fields solely represent the propagated tropospheric delay. The velocity distribution of each simulation can be evaluated visually using histograms, as shown in Figure 5b,e,h. Because each velocity solution depends on a set of randomly selected multipliers, we conducted 100 simulations for each temporal coverage ranging from 3 years to 10 years. Instead of visually analyzing all the resulting velocity fields, we stitched 100 velocity fields of the corresponding temporal span and analyzed the resulting histogram (Figure 5c,f,i).

The histogram of stitched velocity fields represents a general distribution of propagated errors in the time series. We used the joined distributions and calculated the extent of the 95% confidence interval. We presented the resulting propagated error values as box plots (Figure 5c).

## 4. Results

We applied our methodology to three cases of tropospheric delay; vertical stratification, turbulence mixing, and their combination. The combined case represents a more realistic approach to real-world applications. For each case, we first presented a representative velocity map, which was calculated for a 3-year-long series with a 35-day acquisition interval (Figure 5a,d,g). We also presented the histogram of velocity distributions (Figure 5b,e,h). The histograms of joined velocity fields are presented in Figure 5c,f,i.

For the stratified delay, the velocity field follows the elevation pattern (Figure 3a) and the magnitude of errors vary in the range of ±2 mm/yr (Figure 5a,b). The velocity distribution of a single velocity map mimics the spatial pattern of elevation distribution (Figure 5b) since the input simulated scenes are generated by multiplying the inverted and normalized DEM with random variables. However, the velocity distribution obtained from multiple tests shows symmetric distribution (Figure 5c).

For turbulent delay, the velocity field is random in space and the magnitude of errors varies in the range of +5 mm/yr to −2 mm/yr (Figure 5d). The velocity distribution of a single velocity field shows a normal distribution skewed towards the positive side (Figure 5e). The distribution of joined velocity fields shows a normal distribution centered around zero (Figure 5f).

In the case of combined delay components, the magnitude of errors increases in the positive direction because the sample turbulence delay is skewed towards the positive side (Figure 5h). The spatial pattern of the velocity field shows characteristics of both vertical stratification and turbulence mixing components. The velocity distribution of the sample velocity field shows that the propagated error values skew towards positive more than in the case of solely turbulence mixing. The velocity distribution of joined velocity fields exhibits a normal distribution around zero.

**Figure 6:**
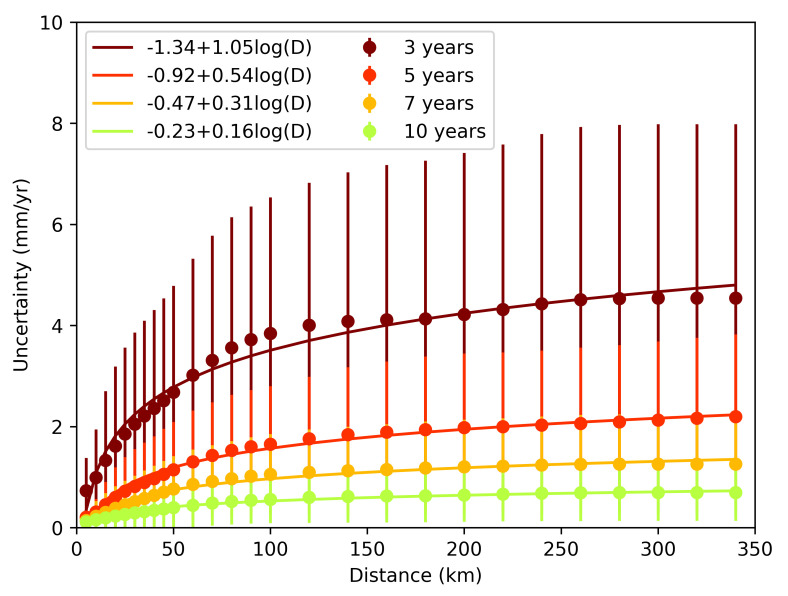
Velocity uncertainties as a function of distance from teh reference point for time-series lengths 3, 5, 7, and 10 years.

The uncertainties of the InSAR-derived velocity map are strongly affected by the tropospheric delay and increase with distance from the reference point. Our simulations provide an excellent opportunity to quantify uncertainty level change as a function of distance. We conducted this analysis by calculating the variance in the velocity fields for each time-series length and by summing the variances of corresponding time-series lengths. After calculating the average variance, the standard deviation of each pixel was calculated. To capture the change of uncertainties with distance, we calculated the uncertainties from a reference point with a 5 km interval between 0 and 50 km, 10 km interval between 50 and 100 km, and 20 km interval beyond 100 km. The uncertainties change as a monotonic, nonlinear increase, possibly logarithmic as the distance from the reference point increases, which is in agreement with previous studies, until they flatten out at a distance, beyond which the noise is uncorrelated. (e.g., [10,15]) (Figure 6). We calculated the best fitting logarithmic function using the following equation:

(5) σ=A+B×log(x)

where σ is the uncertainty and *x* is the distance to the reference point. *A* and *B* are the best-fitting parameters calculated using the least squares method.

**Figure 7:**
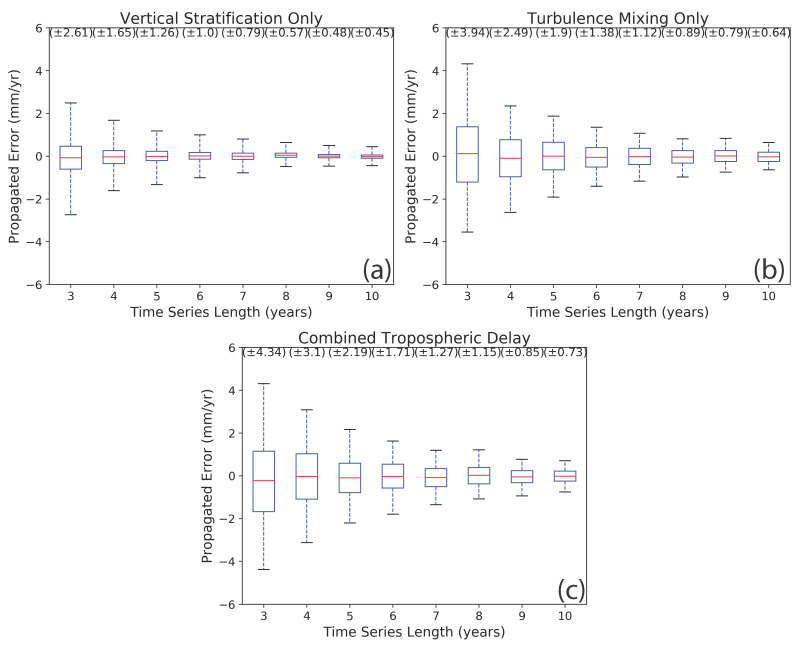
Velocity distributions as a function of time-series length for a 35-day acquisition interval: the blue boxes show the 68% confidence interval, the red lines represent the mean, and the extent of the whiskers shows a 95% confidence interval of the distribution. The values in brackets on the top show the calculated 2σ distribution of the propagated error in millimeter per year for each time-series length. (**a**) The distribution of propagated error with vertical stratification phase delay only, (**b**) the distribution of propagated error with turbulence mixing delay only, and (**c**) the distribution of propagated error in the case of the combination of both phase delays.

We present the results of our simulation tests using box plots (Figure 7). The box plots were calculated from the histogram of joined velocity fields and represent the 95% confidence limit distribution of propagated error for temporal coverage. In the case of vertical stratification, the box plot shows deviations varying from ±2.61 mm/yr at a 3-year time-series length to ±0.45 mm/yr at a 10-year time-series length (Figure 7a). Turbulence mixing has higher distribution values than vertical stratification, with values ranging from ±3.94 mm/yr at 3-year temporal coverage to ±0.64 mm/yr at a 10-year time-series length. When we combined both components, the distribution of propagated errors displays propagated error from ±4.34 mm/yr at 3 years temporal coverage to ±0.73 mm/yr at 10 years time-series length (Figure 7c). Even though the combined delay is simulated by adding vertical stratification and turbulence mixing, the results do not exhibit the sum of the vertical stratification and turbulence mixing cases. Our results are specific to the study area of Socorro Magma Body because they were calculated using site-specific parameters. However, the overall trend of uncertainty decay with temporal coverage applies to all study areas.

## 5. Sensitivity Analysis

The results presented above were obtained with three parameters, namely, range of standard deviation of vertical stratification, magnitude range of turbulence mixing, and acquisition interval. To evaluate the relative contribution of each parameter to the propagated errors, we conducted sensitivity studies in which each parameter was varied systematically. We conducted these studies on both delay components but present only the results of the combined delay, which is the more realistic case.

**Figure 8:**
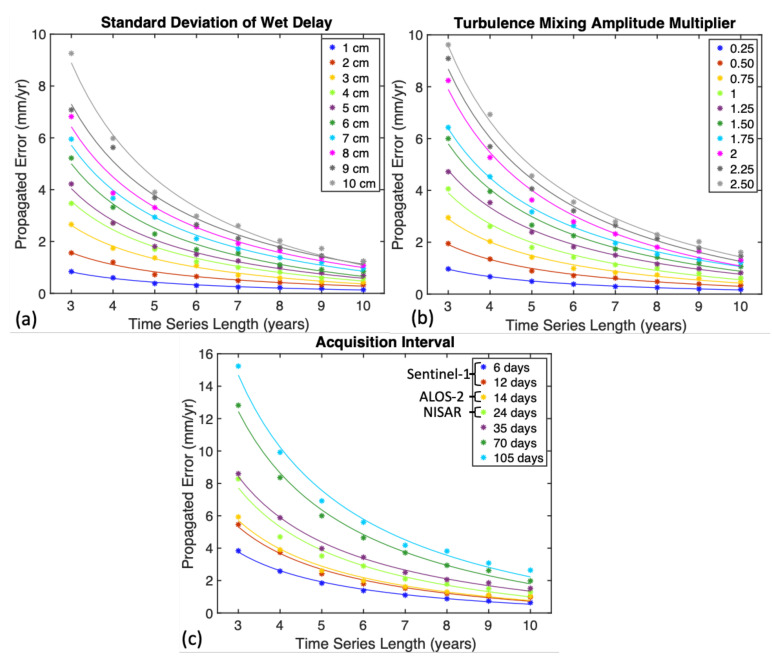
Sensitivity of the estimated detection thresholds with respect to assumed parameters: (**a**) the variation in standard deviation of wet delay calculated from MODIS PWV; (**b**) the scaling parameter for *n* in Equation (4); and (**c**) the variation in detection thresholds with acquisition intervals of 6, 12, 24, 35, 70, and 105 days. The best-fitting 1/T trends are plotted using the same color scale.

Thus far, our simulations assumed that the vertical stratification component of the tropospheric delay varies with a 3 cm standard deviation, which is based on MODIS observations from our semiarid study area. However, in a more humid climate, as in the tropics and subtropics, the range of tropospheric delay can be higher. Thus, we repeated the same simulations varying the parameter in the range of 1–10 cm (Figure 8a). Our results indicate a linear dependency of the propagating error on the delay range parameter. For example, the detection threshold for a 10-year-long time-series in the tropics (delay range ⩾ 10 cm) with the topography of our study area is 2 mm/yr, which is about three times larger than our semiarid study area (delay range 3 cm) (Figure 8a).

The second sensitivity test was carried out to evaluate the input of turbulence mixing. We introduced a new multiplier that varied between 0.25 and 2.50 to represent different climatic conditions. For example, a small multiplier (<1) reflects the less humid (more arid) condition of the troposphere than in our study area in New Mexico, which is characterized by a semiarid condition; a large multiplier (>1) reflects more humid conditions, as in the tropics. This multiplier controls the range of the simulated turbulence mixing amplitude and was not introduced during our initial simulations (i.e., a value of 1). The variation of turbulence mixing resulted in a similar linear behavior to that in vertical stratification. Our results indicate a linear dependency and vary from 1 mm/yr to 10 mm/yr for 3-year-long time series (Figure 8b).

The last sensitivity test evaluated the contribution of the acquisition interval on the propagated errors. In our previous simulations, we used a 35-day interval, which reflects the acquisition intervals of ERS-1/2 and Envisat satellites. Here, we evaluated the effect of shorter and longer acquisition intervals reflecting new acquisition capabilities of Sentinel-1, ALOS-2, and upcoming NASA-ISRO Synthetic Aperture Radar (NISAR) satellites. We simulated the effect of tropospheric delay with acquisition intervals of 6, 12, 24, 35, 70, and 105 days. In these simulations, we kept the range delay parameters 3 cm for vertical stratification and 1 for turbulence mixing. The results of our simulations indicate that the effect of the tropospheric delay on the detection threshold of the InSAR time series is lower in shorter acquisition intervals. For example, the 105-day acquisition interval and 3-year-long time series has a detection threshold (≈15 mm/yr) of about three times larger than the 6-day acquisition interval for the same time-series length (≈4 mm/yr) (Figure 8c).

## 6. A Case Study: Application to Socorro Magma Body

### 6.1. Study Area

**Figure 9:**
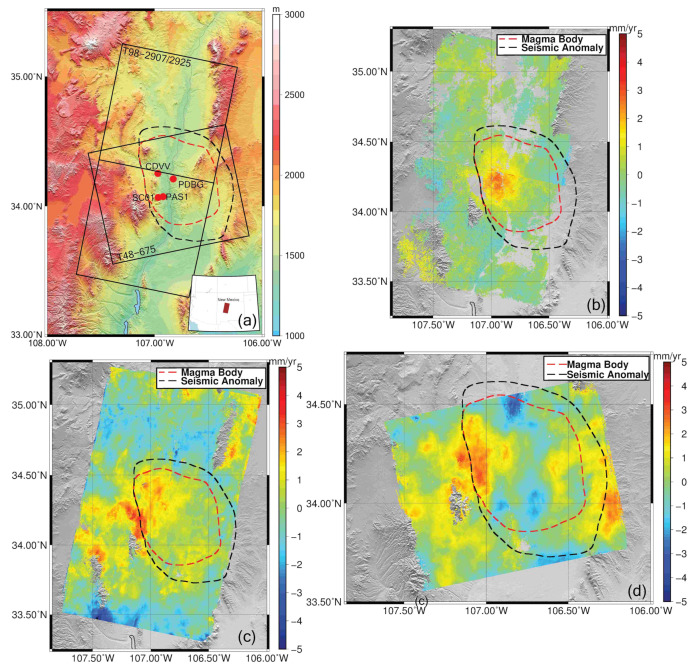
(**a**) The ERS and Envisat satellites share the same track and frame numbers (98-2907/2925) in descending orbit. The ascending orbit of the Envisat satellite covers the study area with frame 675 of track 48. The black dashed line represents the so-called Socorro Seismic Anomaly and the red dashed line represents the Socorro Magma Body defined by [47]. Deformation rates were calculated over Socorro Magma Body (SMB) by using ERS-1/2 and Envisat data. (**b**) The deformation rate map derived from frames 2907 and 2925 of descending track no. 98 of the ERS-1/2 data acquired between 1992–2007. (**c**) The deformation rate derived from Envisat descending scenes of the same track and frame acquired between 2006–2011. (**d**) The deformation rate map derived from Envisat frame 675 of ascending track no 48.

Socorro Magma Body (SMB) is located in the Rio Grande Rift, central New Mexico (USA), and is one of the largest and deepest magma intrusions on Earth (Figure 9a) [45]. SMB was first identified by seismic studies as a region of high seismicity, the so-called Socorro Seismic Anomaly [46,47]. Identification of a region of melt in the mid-crust explained the surface uplift in the area [48,49]. Seismic sounding and microearthquake studies revealed the existence of a seismic reflector about 50–70 km wide at the depth of 19 km [50,51]. However, uplift of the area was observed before the existence of an intrusion was discovered. Three leveling surveys over the area conducted in 1911, 1951, and 1980–1981 showed an uplift rate of a few millimeters per year [49].

The advancement of the InSAR technique provided scientists with a new method to observe the deformation of SMB on a larger scale. Fialko and Simmons (2001) noted an uplift rate of 2 mm/yr using ERS data acquired between 1992 and 1999 [40]. Finnegan and Pritchard (2009) extended the time series to 2006 and calculated a similar velocity of 2.5 mm/yr [52]. The time series was extended by including images from 2000 and 2006 in the network. Pearse and Fialko (2010) used the same approach and noted a velocity of 2.2 mm/yr, similar values to those found in previous studies [45].

Campaign mode and continuous GPS studies have been carried out on SMB, confirming the continuous uplift of the area. The authors of [53] reported that three campaigns were carried out in 2002, 2003, and 2005, revealing an uplift of 20 mm in 2002. EarthScope’s Plate Boundary Observation (PBO) has four GPS stations deployed on and around the SMB (Figure 9a) [54,55,56,57]. Stations PAS1 and SC01 started collecting data in 2001 and are still active today. However, station PAS1 does not have a continuous time series. The CDVV and PDBG stations started collecting data in November 2005 and stopped in October 2013, providing a complete time series over this time interval. The GPS data confirm that the uplift over SMB is continuous and present during the time of InSAR acquisitions [58].

### 6.2. Data and Processing

We used InSAR acquisitions from the ERS-1, ERS-2, and Envisat satellites. The descending orbits of ERS-1 and ERS-2 cover the study area with frames 2907 and 2925 of track 98. The descending orbit of the Envisat satellite also shares the same track and frame numbers with the ERS satellites; however, the ascending orbit of Envisat covers the study area with frame 675 of track 48 (Table 1).

We use a standard processing chain for our InSAR data sets. Zero Doppler single look complex (SLC) data were generated using the Modular SAR Processor (MSP) of Gamma Remote Sensing software [59]. We use ROI_PAC software of Caltech/Jet Propulsion Laboratory (JPL) to generate our interferograms [60]. Interferometric pairs were selected by constraining temporal and perpendicular baselines. The perpendicular baseline between two SAR images was chosen to be smaller than 250 m, while the temporal baseline was forced to be between 300 days and 3500 days. The selection of temporal baseline constraints was decided by considering the amplitude of the deformation signal and temporal coverage of the study area. Previous studies over the area suggest that the deformation rate is between 2 to 2.5 mm/yr. We chose a minimum temporal baseline that is almost 1 year to increase the signal-to-noise ratio (SNR) and a maximum temporal baseline to have a connected network in time so the design matrices for the time-series inversion have full rank. The Shuttle Radar Topography Mission (SRTM) 1 arc-second digital elevation model was used to remove the topography-related phase contribution from each interferogram [61]. The statistical-cost network-flow algorithm for phase unwrapping (SNAPHU) was used to unwrap the interferograms after coregistering the whole network to a single master interferogram [62]. All phase unwrapped interferograms were spatially referenced to the same coherent pixel, and phase unwrapping errors were corrected by applying a phase-closure technique [8,63,64]. The SBAS method was adopted to obtain the phase history of each pixel [21], and then, the stratified tropospheric delay was corrected using North American Regional Reanalysis (NARR) data [29,30]. Errors due to topography were corrected using the approach defined in Fattahi and Amelung (2013) [5]. In our Envisat data sets, we corrected for the Local Oscillator Drift (LOD) as an additional step carried out by utilizing an empirical method that adjusts the range change history for each pixel [65]. A temporal coherence constraint of 0.7 was imposed for the selection of coherent pixels to produce our deformation maps (Figure 9) [66].

The ERS data set has 15 years of temporal coverage with acquisitions from 1992 to late 2006. The estimated deformation rates from the ERS data set reaches a maximum of 2.5 mm/yr, which is in agreement with previous studies [40,45,52] (Figure 9b).

Even though the estimated deformation rates from descending tracks of the ERS and Envisat satellites agree, the Envisat results are mainly dominated by tropospheric noise (Figure 9c,d). We analyzed Envisat’s medium resolution imaging spectrometer (MERIS) data and calculated the wet delay component of tropospheric delay along with the cloud coverage [26]. After dropping images that exhibited more than 20% cloud coverage, the MERIS data set was smaller than our InSAR data set. Partial corrections only introduced more artifacts to our results, so MERIS data was not used for correction.

## 7. Discussion

In this study, we developed a simulation technique to systematically investigate the impact of tropospheric phase delay on InSAR time series by accounting for two main tropospheric processes: vertical stratification, and turbulence mixing. We demonstrated the usefulness of our technique by using regional specific parameters derived for SMB in the southwestern US, which is characterized by a moderate relief (2000 m) and semiarid climate. Our results for this area revealed that the uncertainty levels of the vertical stratification component are 1–5 mm/yr, of the turbulence mixing is 1.5–8 mm/yr, and of the combined components is 1.5–9 mm/yr, in which the ranges reflect the results obtained with different time-series lengths. These results reveal that vertical stratification has a significantly smaller impact on time-series uncertainty level than turbulence mixing. Seventy to ninety percent of the uncertainty arises from the turbulence mixing component. Furthermore, our results emphasize the importance of long time series, which reduces the uncertainty level for the combined component analysis from 8 mm/yr for a 3-year long series to 1.5 mm/yr for a 10-year long series. The 2 mm/yr uncertainty threshold, which is the uplift rate of SMB, occurs with a 7-year and longer time series. Since we simulated time-series epochs, our results are independent of frequency. The effect of tropospheric delay is observed as fringe interferograms. Different sensors operating at different wavelengths (e.g., Sentinel-1 with C-band and ALOS-2 with L-band) exhibit different numbers of fringes in interferograms.

The effect of the troposphere on InSAR continues to be discussed extensively in the literature. Previous studies have shown that the systematic vertical stratification component of the troposphere can be removed using tropospheric models (e.g., [29,30]). Jolivet et al., 2014, reported the results of a case study in northern Chile, where the data set extended from the coast (low elevation) in the west to mountains (high elevation) in the east. The estimated tropospheric delay was correlated with the topography and reached up to a few centimeters. The dominant driver of the tropospheric delay came from turbulence mixing at lower altitudes [30]. Fattahi and Amelung (2015) analyzed the systematic and stochastic components based on MODIS precipitable water vapor observations and atmospheric models and reported variations in the troposphere between 5 and 10 cm along the western India plate boundary [10]. Liao et al., 2020, reported that adapting the correction method described by Yu et al., 2018, which incorporates data from GPS observations and numerical weather models, improved their results by 13% [67,68]. Both of these studies suggest that adopting proper tropospheric correction methods for the study area and data set can improve the time-series results. Our results exhibit a systematic and localized vertical stratification effect, which is in line with the findings of the previous studies. This agreement is achieved by the use of topographic information on the study area and parameters obtained from MODIS PWV data. The high number of repeated simulations for each time-series length ensures the statistical significance of our results.

A comparison of velocity uncertainties and the distance to the reference pixel shows that the uncertainties increase with distance. Emardson et al. (2003) showed the relationship between InSAR uncertainties and distance to reference point. Our analysis of change in uncertainties with distance is in agreement with the findings of Emardson et al. (2003) [15]. However, we do not use this information while reporting our results because we are calculating the distribution of values in estimated velocity fields. We concatenated the estimated velocity fields to generate one large matrix that includes all velocity fields with the corresponding temporal coverage and then analyzed its histogram to estimate an expected detection threshold within a confidence limit. Our results show that there is a logarithmic relationship between distance and uncertainties.

Our results reveal that the relationship between propagated error and time-series length exhibits the characteristic shape of 1/T decay. This relationship seems very similar to the relationship of white noise and time span of data in GPS studies. Zhang et al. 1997, suggested that the relationship can be denoted as follows:

(6) $$\sigma_{WN}=\frac{a_{WN}}{T}\sqrt{\frac{12(N-1)}{N^{2}+N}}$$

where $\sigma_{WN}$ is the velocity uncertainty, $a_{WN}$ is the magnitude of the white noise, T is the total observation interval, and N is the number of observations [69]. The equation suggests that the white noise of an overdetermined system is proportional to the time span of the data set and the number of data points. In our case, the decay rate seems to exhibit a 1/T decay characteristic in agreement with Equation (6). The decay of the detection threshold estimates follows the same pattern regardless of any parameter introduced in our simulations. This represents the correlation between the number of images used in a study and the expected detection threshold. As seen in our sensitivity analysis with different acquisition intervals, larger data sets have smaller detection thresholds (Figure 8). We can use our detection threshold estimates to solve Equation (6) for the magnitude of the white noise ($a_{wn}$), as shown in Table 2. Our results indicate similar values of white noise magnitudes in the range of 4.3–5 cm, which is comparable to the magnitude of the turbulent component of the tropospheric delay. Since the dominant impact on the combined case comes from turbulence mixing, the calculated white noise levels are similar to turbulence mixing levels.

Recent studies revealed that tropospheric phase delay has a significant seasonal component (e.g., [10]). Despite the importance of the seasonal component, we did not consider seasonality because our simulated time-series lengths range from 3 to 10 years. The length and temporal resolution of our data sets allow us to assume that the effect of seasonality can be ignored. However, a network of intermittent acquisitions will be affected by the seasonality of tropospheric delay. Another limitation of our method is the assumption of a linear relationship between topography and vertical stratification. This linear relationship between topography and vertical stratification has been shown and used in previous studies (e.g., [3,16,37]) but does not necessarily apply to all real-world cases (e.g., [16,70,71]).

## 8. Conclusions

We analyzed error propagation through InSAR time-series products due to systematic and stochastic components of the troposphere and their combination. A novel approach that adopts simulations was presented. This approach is based on modeling each component individually and on running 100 tests for time-series lengths from 3 years to 10 years. We present the results of the propagated error for individual cases of vertical stratification, turbulence mixing, and the combined influence of both components specific to our semiarid study area, Socorro Magma Body. The findings of this study are as follows:

(1) Tropospheric delay due to vertical stratification is a systematic error source that produces localized errors around high topographic gradients. We found that a data set length of 6 years (given an acquisition interval of 35 days) is required to achieve a ≈1 mm/yr detection threshold.
(2) Tropospheric delay due to turbulence mixing is a stochastic error and cannot be removed by modeling in space. Turbulence mixing has a larger impact on time-series products than vertical stratification. We showed that a 7-year (or longer) data set with a 35-day acquisition interval is required to achieve a ≈1 mm/yr detection threshold over 50 km.
(3) By simulating the combined effect of both vertical stratification and turbulence mixing, we retrieved errors of similar magnitude to our simulations of turbulence mixing alone. Significantly, this highlights that turbulence mixing represents the main source of tropospheric errors in real-world applications. As such, even if we can model and systematically remove errors due to vertical stratification, nonnegligible errors may persist. A ≈1 mm/yr detection threshold would be possible with a time series longer than 8 years with a 35-day acquisition interval.
(4) The decay characteristics of propagated errors concerning temporal coverage exhibit 1/T decay, which is denoted for the GPS studies by Zhang et al. (1997).
(5) The acquisition strategies of new-generation Sentinel-1 satellites with a 6-day acquisition interval will provide ≈1 mm/yr detection level beyond 5 years with a 6-day acquisition interval.
(6) We cannot quantitatively distinguish between the tropospheric delay and the slow uplift signal over Socorro Magma Body with a 5-year-long Envisat time series with the current methods. Our results show that a data set longer than 8 years is required with a 35-day acquisition interval. The ERS data set with 15-year-long time series fulfills this requirement and provides a high-resolution deformation map that is minimally affected by the tropospheric delay.

## Figures and Tables

**Table 1:** Summary of the Synthetic Aperture Radar (SAR) data sets used in this study.

Satellite	Flight Direction	Track	Frame	No. of Images
ERS-1/2	Desc.	98	2907	33
ERS-1/2	Desc.	98	2925	38
Envisat	Desc.	98	2907, 2925	27
Envisat	Asc.	48	675	22

**Table 2:** Estimated white noise in the case of a combination of vertical stratification and turbulence mixing components (Figure 7c), with the formula (Equation (6)) described by Zhang et al. (1997) [69], where T is the time-series length in number of years, $\sigma_{wn}$ is the detection threshold taken from Figure 7c, N is number of images, and $a_{wn}$ is the estimated white noise.

T (Years)	$\sigma_{\mathrm{wn}}$ (mm/yr)	N (# of Images)	$a_{\mathrm{wn}}$ (cm)
3	8.68 (±4.34)	32	4.39
4	6.20 (±3.10)	42	4.75
5	4.38 (±2.19)	53	4.69
6	3.42 (±1.71)	63	4.78
7	2.54 (±1.27)	73	4.45
8	2.30 (±1.15)	84	4.93
9	1.70 (±0.85)	94	4.33
10	1.46 (±0.73)	105	4.36

**Figure A1:**
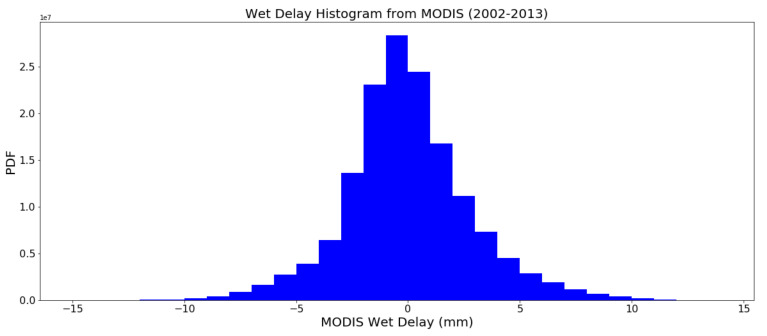
Histogram of wet delay generated from moderate resolution imaging spectroradiometer (MODIS) observations between 2002 and 2013: the data set consists of wet delay calculated over our study area. The histogram shows that the wet delay values are distributed normally around 0 with 3 cm standard deviation.

## Data Availability

Data sharing not applicable.

## Appendix A. Derivation of Deviatoric Tropospheric Phase Delay

Synthetic Aperture Radar (SAR) phase measures the Line of Sight (LOS) range between the satellite and the Earth’s surface, which is also contaminated by tropospheric delay. The actual phase (ϕ) for a single acquisition is defined as follows:

(A1) $$\phi =\frac{2\pi }{\lambda }2R=\frac{4\pi }{\lambda }R$$

where ϕ is the phase, *R* is range, and λ is the radar wavelength. The range is calculated from the one-way travel time of the radar signal (*t*/2) multiplied by the speed of light ($R=C_{0}*\frac{t}{2};C_{0}$ is speed of light). Since the speed of light is delayed when traveling through the troposphere, the range measurement is not an accurate description of the true LOS distance. Thus, the phase measurement represents LOS distance, atmospheric delay, surface deformation, and other sources, which are considered as noise:

(A2) $$\phi =\phi_{los}+\phi_{trop\_delay}+\phi_{iono\_delay}+\phi_{topo}+\phi_{orb}+\phi_{defo}+\phi_{noise}$$

where $\phi_{los}$ is the true distance from the satellite to the surface, $\phi_{trop\_delay}$ is the additional distance due to tropospheric delay, $\phi_{iono\_delay}$ is the delay caused by ionosphere, $\phi_{topo}$ is the phase due to DEM errors, $\phi_{orb}$ is the orbital error due to perpendicular baseline, $\phi_{defo}$ is the surface deformation, and $\phi_{noise}$ is the measurement noise, which includes all other sources. When using a stack of N acquisitions and assuming that tropospheric delay is the only noise in the phase measurement ($\phi_{noise}=0$) and that the study area is free from deformation ($\phi_{defo}=0$), the mean phase can be described as follows:

(A3) $$\phi_{mean}=\phi_{mean\_los}+\phi_{mean\_trop\_delay}$$

The term $\phi_{los}$ is defined as the true distance from the satellite, which varies from one acquisition to another based on the satellite’s orbit. For simplicity, in this study, we assume that all acquisitions were obtained from the exactly same position in space. Based on this assumption, $\phi_{los}$ is equal in all acquisitions. In reality, $\phi_{los}$ varies from one acquisition to another, reflecting shifts in satellite orbits. However, precise orbit estimations provide sufficient data to correct the $\phi_{los}$ offset between the various acquisitions. On the other hand, $\phi_{trop\_delay}$ varies for each acquisition, and its meaning is defined as follows:

(A4) $$\begin{array}{c} \phi_{mean\_trop\_delay}=\frac{1}{N}\sum_{1}^{N}\phi_{trop\_delay_{i}}; \end{array}$$

The phase in each acquisition can be described in terms of its deviation from the mean:

(A5) $$\phi_{i}=\phi_{mean}+\phi_{dev_{i}}$$

where

(A6) $$\phi_{dev_{i}}=\phi_{los}+\phi_{mean\_trop\_delay}-\phi_{dev\_trop\_delay_{i}}$$

Since the actual SAR phase measurement only consists of the non-integer portion of the theoretical phase (ϕ), the actual range between the surface and the satellite cannot be estimated. However, repeat pass SAR interferometry, which detects phase changes between two acquisitions collected roughly from the same location, provides an accurate measurement of relative distance changes. Thus, we perform interferometric calculations by subtracting the phase values between two acquisitions:

(A7) $$\begin{array}{c} \phi_{int_{i,j}}=\phi_{i}-\phi_{j}=\left(\phi_{mean}+\phi_{dev_{i}}\right)-\\ \left(\phi_{mean}+\phi_{dev_{j}}\right)=\phi_{dev_{i}}-\phi_{dev_{j}} \end{array}$$

Substituting Equation (A6) into Equation (A7):

(A8) $$\phi_{int_{i,j}}=\phi_{dev\_trop\_{delay}_{j}}-\phi_{dev\_trop\_{delay}_{i}}$$

This derivation indicates that, in the case where the tropospheric delay is the sole error source (with no surface deformation), the interferometric phase can be calculated by the difference between the deviatoric tropospheric phase delay of any two given acquisitions.

The deviatoric tropospheric phase delay, which is in units of radians, can be converted into a length unit by multiplying the phase by any given sensor wavelength.

(A9) $$dev\_tropospheric\_delay_{j}=\phi_{dev\_trop\_delay_{j}}\times \lambda$$

(A10) $$\phi_{dev\_trop\_delay_{j}}=dev\_tropospheric\_delay_{j}/\lambda$$

## Appendix B. Absolute Standard Deviation of Wet Delay

We used a total of 2939 MODIS acquisitions over the study area to calculate the standard deviation of wet delay between 1 January 2002 and 31 December 2012 (Figure A1). The resulting histogram is a 0 mean, 3 cm standard deviation normal distribution. We analyzed the normality of the histogram using the Shapiro–Wilk test [72] and found that the data represent a Gaussian distribution.
